# The Next Chapter in Cancer Diagnostics: Advances in HPV-Positive Head and Neck Cancer

**DOI:** 10.3390/biom14080925

**Published:** 2024-07-30

**Authors:** Antea Krsek, Lara Baticic, Tamara Braut, Vlatka Sotosek

**Affiliations:** 1 Faculty of Medicine, University of Rijeka, 51000 Rijeka, Croatia; tea.krsek@gmail.com; 2 Department of Medical Chemistry, Biochemistry and Clinical Chemistry, Faculty of Medicine, University of Rijeka, 51000 Rijeka, Croatia; 3 Department of Otorhinolaryngology and Head and Neck Surgery, Clinical Hospital Centre Rijeka, 51000 Rijeka, Croatia; tamara.braut@uniri.hr; 4 Department of Anesthesiology, Reanimatology, Emergency and Intensive Care Medicine, Faculty of Medicine, University of Rijeka, 51000 Rijeka, Croatia; vlatkast@uniri.hr; 5 Department of Clinical Medical Sciences I, Faculty of Health Studies, University of Rijeka, 51000 Rijeka, Croatia

**Keywords:** biomarkers, head and neck squamous cell carcinoma, HPV-16, human papillomavirus, oropharyngeal squamous cell carcinoma, p16

## Abstract

Human papillomavirus (HPV)-associated head and neck squamous cell carcinoma (HNSCC), particularly oropharyngeal squamous cell carcinoma (OPSCC), is an increasingly prevalent pathology worldwide, especially in developed countries. For diagnosing HPV in HNSCC, the combination of p16 immunohistochemistry (IHC) and polymerase chain reaction (PCR) offers high sensitivity and specificity, with p16 IHC being a reliable initial screen and PCR confirming HPV presence. Advanced techniques like next-generation sequencing (NGS) and RNA-based assays provide detailed insights but are primarily used in research settings. Regardless of HPV status, standard oncological treatments currently include surgery, radiation, and/or chemotherapy. This conventional approach does not account for the typically better prognosis of HPV-positive HNSCC patients, leading to increased chemo/radiation-induced secondary morbidities and reduced quality of life. Therefore, it is crucial to identify and detect HPV positivity and other molecular characteristics of HNSCC to personalize treatment strategies. This comprehensive review aims to summarize current knowledge on various HPV detection techniques and evaluate their advantages and disadvantages, with a focus on developing methodologies to identify new biomarkers in HPV-positive HNSCC. The review discusses direct and indirect HPV examination in tumor tissue, DNA- and RNA-based detection techniques, protein-based markers, liquid biopsy potentials, immune-related markers, epigenetic markers, novel biomarkers, and emerging technologies, providing an overall insight into the current state of knowledge.

## 1. Introduction

High-risk human papillomavirus (HR-HPV) infection can cause malignant tumors to grow in a variety of squamous tissues across the body, including the areas of head and neck, especially the oropharynx (comprised of several tissues and structures, including the soft tissue, the base of the tongue, palatine tonsils, the posterior pharyngeal wall, and the back wall of the throat), all of which have the ability to spread to nearby subsites [1]. Globally, 100,500 new cases of HPV-associated oropharyngeal cancer (HPV-OPC) were reported in 2018 [2]. Of these, 35–80% of cases were shown to be prevalent in western nations [3]. HPV-related head and neck squamous cell carcinoma (HNSCC), in particular oropharyngeal squamous cell carcinoma (OPSCC), has become more common in both Europe and the US. This trend has been emphasized in various studies, reporting that in specific sites, HPV is linked to 38–80% of OPSCCs and 30% of HNSCCs [4,5,6]. However, oropharyngeal cancer (OPC) in Europe and the USA has multiple etiologies beyond HPV infection [3]. Tobacco use is a predominant risk factor for OPC. Carcinogens in tobacco smoke induce mutations in the DNA of oropharyngeal cells, promoting cancer development. Chronic and heavy alcohol consumption is another significant risk factor. Alcohol serves as an irritant, making oropharyngeal tissues more vulnerable to carcinogens. The combined use of alcohol and tobacco synergistically elevates the risk of OPC. Dietary insufficiencies, particularly diets low in fruits and vegetables that are rich in essential vitamins, minerals, and antioxidants, may heighten the risk of developing OPC. A family history of cancers, including OPC, suggests a genetic susceptibility, increasing the likelihood of the disease. Exposure to specific workplace chemicals and pollutants, such as asbestos, formaldehyde, and other industrial chemicals, can elevate the risk of OPC. The lack or inadequacy of screening programs can delay OPC diagnosis. Early detection is critical for favorable treatment outcomes. In addition, lower socioeconomic status is correlated with a higher risk of OPC due to factors such as limited healthcare access, higher prevalence of tobacco and alcohol use, poor nutrition, and reduced availability of preventive measures and education. Finally, while HPV is a well-known risk factor, certain sexual behaviors, including having multiple sexual partners and engaging in oral sex, increase the risk of OPC due to a higher likelihood of acquiring infections and other sexually transmitted diseases [1,4,5,6,7,8].

It was believed for many years that the causes of all OPSCC instances were the same. But in 1983, a portion of OPSCC tumors were shown to express HPV antigens [7]. Following further research, we now have an improved comprehension of this disease mechanism and by that we can classify HPV-positive(+)OPSCC as a different disease process from HPV-negative(−)OPSCC, with different genetic, epidemiological, and predictive characteristics [8].

At the cellular level, the tumor microenvironments (TMEs) of HPV-induced OPSCC are well-differentiated from the attracted immune cell infiltrates in contrast to HPV-negative OPSCC [9]. Differences in the immune landscape are a defining characteristic of HPV+HNSCC. For example, immune signatures and immune landscape predict the treatment response, which was recently summarized by Yarbrough et al., discussing the potential for biomarkers to revolutionize personalized therapy [10]. It is important to mention that PIK3CA and FGFR pathway mutations, as well as overexpression of CDKN2A, which codes for p16, have been found in HPV+OPSCC in genetic studies [1]. Additionally, patients with HPV+OPSCC have distinct clinical characteristics. These patients usually present at an earlier age with a background of not smoking or drinking alcohol and manifest with adenopathy and with unknown primary tumors [11,12].

A further important difference lies in the fact that HPV+OPSCC is more responsive to existing standard treatments, particularly non-surgical treatment options, compared with its HPV−hollow counterpart, and has a better outcome regardless of stage [1]. Thus, the 8th edition of the AJCC TNM staging system acknowledged this difference and characterized HPV+OPSCC as an unique medical condition [13].

Although progress has been made, there are still no individualized treatment plans for HPV+OPSCC. Regardless of HPV status, surgery, radiation, and/or chemotherapy are currently used as standard treatments. This approach ignores the better prognosis that HPV+OPSCC patients usually have, which increases morbidity and puts younger patients at risk of radiation-induced secondary cancers by 15–40% [14]. Trials for radiotherapy de-escalation are being conducted to see if lowering the dose in HPV+OPSCC patients can keep their cure rates high, maintain their quality of life, and reduce their risk of developing secondary tumors. Although first findings are encouraging, comparison is challenging because of different designs, limited sample numbers, and other difficulties [15]. In spite of this, research continuously demonstrates that de-escalation is both safe and practicable, which helps to customize HPV+OPSCC treatment [16]. However, for patients who smoke heavily or consume large amounts of alcohol, dosage de-escalation is not advised [17,18].

## 2. Direct HPV Examination in Tumor Tissue

Head and neck cancers (HNCs) can be assessed for HPV-related disease using both direct and indirect methods. Direct methods involve detecting HPV DNA or mRNA expressed in tumor tissues, while indirect methods entail identifying p16Ink4a (p16) expression through immunohistochemistry (IHC). Detecting p16 overexpression serves as a surrogate marker for HPV-related oncogenesis, while the dual-stain cytology combines p16 IHC with Ki-67 staining to identify proliferating cells, enhancing the specificity of p16 as a marker for active HPV infection. The latest knowledge emphasizes the importance of liquid biopsy approaches using ctDNA for non-invasive detection and monitoring of HPV-associated HNCs. These techniques can detect HPV DNA fragments in the bloodstream, potentially enabling early diagnosis and assessment of treatment efficacy [15,16,19,20,21,22,23].

### 2.1. DNA-Based Detection

#### 2.1.1. Polymerase Chain Reaction (PCR)

Polymerase chain reaction (PCR) is an important molecular tool used in diagnosing and differentiating HPV+HNSCC and OPSCC. PCR targets specific sequences of HPV and enhances amplification of its genomes on tissues from tumors, making it easier to quantify the viral load present. In HPV-positive HNSCC and OPSCC, general PCR is used to confirm the detection of high-risk genetic variants, with HPV 16 being the most prevalent [19,20].

PCR-based assays have several advantages for preferable sensitivity and specificity in HPV-related cancer diagnosis. They offer high sensitivity, whereby the test can signal the virus’s presence at minimal levels within the tumor tissues [21,22]. PCR also permits the analysis of multiple HPV genotypes simultaneously, which is especially beneficial for the comprehensive understanding of HPV’s involvement in HNSCC or OPSCC [19,22]. Furthermore, PCR assays can adapt to various kinds of samples, such as formalin-fixed, paraffin-embedded (FFPE) tissues, fine-needle aspirates (FNA), and oral swab samples, making it a good tool for clinical diagnosis.

PCR has been particularly useful for molecular and biochemical investigations of HPV-positive HNSCC, especially in OPSCC, which plays an important role in tumor initiation and progression. PCR assays with analysis of HPV DNA and viral load contribute to the prognosis of patients according to their HPV status, which plays an important role in determining the malignancy of the tumor. In addition, a PCR-based approach has been employed to examine how exactly HPV interacts with certain genetic changes in tumor cells to promote the development of cancers, thus explaining the oncogenic pathways that HPV triggers in HNSCC and OPSCC [23]. PCR proves to be a useful method while diagnosing HNSCC and OPSCC with a positive HPV status and can also help in better understanding and further treating the disease. Due to its high sensitivity, it is versatile and can also offer molecular details of tumor biology when used in HPV-associated HNSCC, which other methods cannot achieve, making it an essential molecular pathology diagnostic tool [19,20,21,22].

#### 2.1.2. Next-Generation Sequencing (NGS)

Next-generation sequencing (NGS) has transformed the method of studying genomes since it allows us to quickly sequence entire genomes or specific regions, which is a huge improvement over older methods like Sanger sequencing [24]. Another older method, Maxam–Gilbert sequencing, uses radioactive labeling, but it has become outdated because of the hazardous chemicals involved [25]. While Sanger sequencing is still used, NGS is preferred because it performs better and has more advantages over traditional methods [26]. Since their creation, NGS platforms have come a long way, making a once expensive and time-consuming process much more practical for oncologists who specialize in head and neck cancer. NGS is already integrated into clinical practice. A recent survey of 1281 oncologists found that about 75% of them use NGS to guide patient care, even though there are no standardized clinical guidelines yet [27].

Through the last decade, plenty of FDA-approved NGS platforms have been created. There are single-gene tests, like the ones that check for EGFR mutations in non-small cell lung cancer, or BRAF mutations in melanoma. And then there are more comprehensive gene panels, such as the FoundationOne CDx by Foundation Medicine in Cambridge, MA [28,29]. When it comes to HNSCC, most specialists prefer using multigene sequencing platforms over single-gene tests. That way, the identification of multiple actionable targets can be addressed, eliminating the necessity for repetitive biopsies and mitigating the risk of treatment delays [30]. Among different techniques, newer platforms like Oxford Nanopore Technologies (ONT) and Pacific Biosciences (PacBio), which can sequence longer DNA fragments and read entire cDNA in one individual operation, are available today [30,31,32,33,34].

Moreover, NGS has revolutionized the study of HPV-associated oral carcinoma by enabling comprehensive genomic, transcriptomic, and epigenomic profiling. This technology provides detailed insights into the molecular mechanisms driving carcinogenesis and aids in identifying prognostic signatures that can predict clinical outcomes. NGS has uncovered specific mutations and copy number variations associated with HPV-positive oral carcinomas. Commonly affected genes include PIK3CA, TP53, and NOTCH1. It has been shown that specific gene expression signatures, such as elevated levels of E2F target genes, are associated with better patient outcomes. NGS-based methylation profiling has identified differential methylation patterns in HPV-associated oral carcinomas. These genomic alterations can serve as biomarkers for prognosis and potential therapeutic targets [35,36].

Indeed, NGS has made a big impact in various fields. It has revolutionized the study of pathogens and oral microbiota—the second largest community of microbes in our bodies. When it comes to studying oral microbiota, scientists rely on two NGS methods: 16S rRNA sequencing and shotgun sequencing. Shotgun sequencing analyzes the entire microbial genome, giving us insights into metabolic pathways and resistance genes. This comprehensive genomic analysis is a breakthrough in the field of microbiology and even in drug development [26]. Furthermore, NGS has brought much-needed clarity to many uncertainties. Traditional methods like Sanger sequencing and PCR-based assays have their limitations, such as processing volume and sensitivity issues. Sanger sequencing, for example, can only detect mutations if they make up around 20–25% of the gene’s total makeup. And while qRT-PCR is fast and sensitive, it can only identify a handful of known sequences [37,38]. NGS, on the other hand, enables whole-exome sequencing, targeted sequencing, and RNA sequencing of tumors. It is especially effective in dealing with tumor heterogeneity, which is a common challenge in solid tumors like oral squamous cell carcinoma (OSCC) [39,40].

In the past, the cost of NGS panels was a major obstacle. Nevertheless, today, with their increased use in clinical treatments, especially for recurrent or metastatic diseases, the costs have gone down and insurance coverage has improved. Multigene NGS platforms are commonly used in the recurrent/metastatic setting of HNSCC to identify potential candidates for biomarker-driven and combination clinical trials. Some researchers are employing NGS as a routine clinical tool to correlate genomic changes with cancer-related results [41].

### 2.2. RNA-Based Detection

#### 2.2.1. MicroRNAs

Cancer has been linked to microRNAs (miRNAs), non-coding molecules that alter gene expression [42]. They are prognostic indicators for several malignancies, such as hepatocellular carcinoma, lung tumors, and B-cell lymphomas. Since HPV-positive and HPV-negative tumors produce miRNAs differently, these molecules are particularly interesting in HNC [43]. It has been noted that a variety of miRNA signatures improve the risk evaluation of tumors that are HPV-positive and HPV-negative [44,45]. For example, a five-miRNA signature (hsa-let-7g-3p, hsa-miR-6508-5p, hsa-miR-210-5p, hsa-miR-4306, and hsa-miR-7161-3p) was found to be a strong predictor of survival and recurrence in a research study involving HPV-negative HNC patients receiving chemoradiotherapy [44,46].

#### 2.2.2. E6/E7 Detection: Blood- and Saliva-Based HPV Biomarkers and Viral Integration

Blood samples taken decades before the disease’s development can reveal antibodies that target HPV E6 antigens, namely the E6 protein, which has been connected to an elevated risk of developing oropharyngeal cancer [47,48]. HPV16-E6 seropositivity is a very sensitive and specific diagnostic indication for HPV-positive OPSCC at the time of diagnosis [48,49,50]. In most cases, HPV16-E6 seropositivity does not significantly correlate with treatment outcome or recurrence [47]. A recent area of interest concerning HPV-positive HNSCC is HPV’s viral integration into the cellular genome of the host. Viral integration has been linked to heightened transcription levels of E6 and E7, which are associated with the progression of carcinoma in cervical cancers, and similarly, its occurrence has been recognized in head and neck carcinoma; however, its correlation with clinical outcomes remains poorly understood [51]. Existing evidence often presents conflicting results, partly due to variations in methods used to detect viral integration. Although some studies suggest epigenetic upregulation of regions surrounding the integrated HPV genome in the host, the clinical implications of these observations are still being investigated [52].

## 3. Indirect HPV Testing in Tumor Tissue

### 3.1. Protein-Based Markers

#### 3.1.1. p16 Immunohistochemistry (IHC) and p16 Overexpression

The p16 protein immunostaining technique provides a low-cost means of identifying high-risk HPV infections in tissues. P16 serves as a “surrogate” biomarker for HPV status and is considered an indirect indicator of HPV-positive disease in OPSCC. It functions as a cell-cycle regulator and protein that suppresses tumor growth, exhibiting overexpression due to HPV-induced oncogenesis. Immunohistochemical assessment is employed to detect p16, with at least 70% nuclear and cytoplasmic expression indicative of p16 positivity. However, it is crucial to note that p16 overexpression can also occur independent of HPV, particularly in oropharyngeal tissues with high HPV prevalence, resulting in a predictive accuracy in identifying positive cases. Guidelines by the College of American Pathologists recommend p16 testing specifically for oropharyngeal tumor specimens based on its proven utility as an independent prognostic indicator for OPSCC, its ubiquitous accessibility, and consistent performance on specimen samples. Nonetheless, this recommendation applies exclusively to OPSCC, and p16 testing is not advised for other tumor types like neuroendocrine or salivary gland tumors [51].

Approximately 20% of patients exhibiting p16-positive tumors yield negative results for HPV DNA or RNA testing [53]. There are also cases of discordance, wherein patients who test positive for p16 are negative for HPV, and the opposite is the case as well. A recent large-scale study involving 7654 patients revealed that discordance between p16 and HPV status adversely affects the outcome, prompting the authors to advocate for broader indications for direct HPV testing. The overexpression of p16 in HPV-negative tumors seems to occur via distinct mechanisms, necessitating diverse detection techniques and taking into account factors not associated with HPV status. Mehanna et al. reported that when relying solely on p16 immunohistochemistry for HPV status determination, approximately 8% of p16-positive patients could be incorrectly classified as having an HPV-positive tumor. Consequently, while routine evaluation of HPV and p16 testing is advised in OPSCC clinical trials and settings requiring detailed counseling, solely categorizing OPSCC patients based on p16 status is insufficient in standard healthcare practice [54]. It should be emphasized that p16 status is not all that accurate, but it is easy to implement in any laboratory vs. the gold standard of PCR or other nucleic-acid based detection. Nucleic acid sequencing can reveal the HPY type and polymorphisms that may help predict the prognosis. Indeed, HPV genetic variation may be associated with HNC clinical characteristics and may have prognostic value, as recently suggested by Gameiro et al. [55].

#### 3.1.2. Cyclin D1

**Figure 1:**
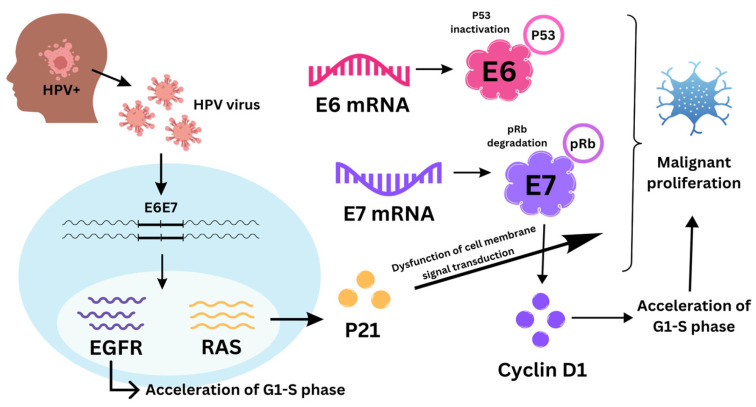
Disruption of the cell cycle caused by human papillomavirus (HPV).

The G1 checkpoint is a critical regulator that controls the course of cell division. It is rigorously controlled by a set of proteins that includes cyclins, CDKs, and CDK inhibitors. Two genes implicated in DNA repair and the cell cycle are cyclin-dependent kinase inhibitor 2A (CDKN2A) and cyclin D1 (CCND1) [56]. According to research, they are known to be a major factor in the proliferation of tumors that can arise in several different anatomical locations, including the esophagus, ovary, breast, colon, lung, and head and neck [57]. In 94% of cases of oral squamous cell carcinoma (OSCC), CCND1 amplification and CDKN2A deletion are present; structural alterations in CDKN2A (such as homozygous deletion and intra- and inter-chromosomal fusions) appear to be frequent [10,56]. Furthermore, research has revealed a homozygous deletion (6%), as well as a mutation (8–12%) in protocadherin FAT1 (FAT atypical cadherin 1) in HNSCC. Furthermore, the Wnt signaling pathway can be activated to promote carcinogenesis by mutation or homozygous deletion of FAT1, leading to functional loss of FAT1 [56]. Prior research also has demonstrated an association between overexpression of cyclin D1 and the development of regional lymph node metastases in HNC, as well as lower rates of disease-free and overall survival [51]. The known mechanisms of disruption of the cell cycle caused by human papillomavirus (HPV) are presented in Figure 1.

#### 3.1.3. Estrogen Receptor Positivity

Recent investigations have explored the potential of tumor estrogen receptor alpha (ERα) positivity as a prognostic marker associated with enhanced survival rate and recurrence-free survival in HPV-positive oropharyngeal cancer [52]. Despite being recognized as a biomarker and therapeutic target in breast cancer, the assessment of ERα in HNSCC has only lately garnered attention. Numerous studies have highlighted a positive outlook for individuals with ERα-positive oropharyngeal squamous cell carcinoma following chemoradiation, even after accounting for different clinical risk factors [58,59]. Although no current studies have delved into ERα as a therapeutic target in head and neck cancer, its presence within tumors may serve as a valuable indicator for treatment selection and de-intensification strategies [60].

## 4. Liquid Biopsy for HPV-Positive HNSCC

**Figure 2:**
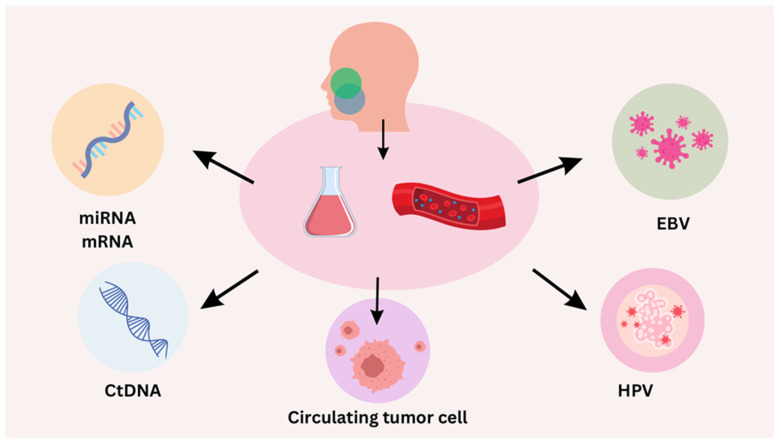
A depiction of liquid biopsy biomarkers (HPV—human papilloma virus; EBV—Epstein–Barr virus; ctDNA—circulating tumor DNA).

Circulating tumor cells (CTCs), cell-free DNA (cfDNA), and other indicators found in the blood are examined during a minimally invasive procedure called liquid biopsy. In order to support diagnosis and treatment monitoring, it can offer real-time information about the genetic composition of tumors, including current HPV status. The main liquid biopsy biomarkers are presented in Figure 2.

### 4.1. Circulating Tumor DNA (ctDNA)

Circulating tumor HPV DNA (ctHPVDNA), which refers to DNA fragments released from tumor cells into the bloodstream, has been identified in approximately 90% of patients with HPV-positive OPSCC using highly sensitive PCR or next-generation sequencing techniques; it may be a promising marker for early cancer detection, treatment monitoring, and detecting recurrence [61]. Unlike traditional biopsy, which is invasive and limited to specific tumor areas, ctDNA analysis, a form of liquid biopsy, offers non-invasive insights into overall tumor burden and gene expression [62]. It exhibits high specificity for malignancy and dynamic variability, influenced by disease burden and treatment response due to its short half-life. ctHPVDNA is currently under investigation given its potential usefulness in diagnosing, treating, and monitoring HPV-positive OPSCC.

ctHPVDNA has been thoroughly investigated in post-treatment monitoring, demonstrating a near-perfect predictive accuracy, with rates exceeding 95–100% for both positive and negative results for disease recurrence and showing potential to enhance surveillance accuracy compared to the current standard of care when combined with PET/CT imaging [63,64,65,66]. Additionally, a tumor tissue modified viral (TTMV) HPV DNA biomarker test seems to predict recurrence of HPV-driven oropharynx cancer after treatment. In a recent study, circulating TTMV HPV DNA was statistically significantly associated with nodal disease at HPV-positive OPSCC diagnosis [64,65].

In the study conducted by Tatsumi et al., a link between ctDNA, particularly HPV 16 DNA (ctHPV16DNA), and quantitative PET parameters in patients diagnosed with HPV-positive head and neck squamous cell carcinoma has been explored. The study involved 50 individuals with oropharyngeal SCC and 5 with SCC of unknown primary tumors, all undergoing pre-treatment blood sampling for ctHPV16DNA assessment and FDG PET-CT scans. Comparative analysis was conducted between ctHPV16DNA levels and various PET parameters like SUVmax, metabolic tumor volume (MTV), and texture features. The findings indicated a moderate association between ctHPV16DNA levels and whole-body MTV, suggesting a potential correlation with tumor burden and heterogeneity observed on PET-CT scans. The results underscored the utility of ctHPV16DNA as a marker for evaluating tumor characteristics in patients with HPV-positive HNSCC [62].

Additionally, changes in ctHPVDNA levels are being investigated for real-time therapy guidance, including dose adjustments during definitive radiation therapy. In patients undergoing surgery, detectable ctHPVDNA is postoperatively associated with a higher risk of residual disease and extranodal extension, suggesting that it serves as a high-risk feature warranting consideration for adjuvant therapy [66,67]. Moreover, ctHPVDNA has been detected in blood samples collected several years before the diagnosis of HPV-positive OPSCC in some patients, indicating its potential use in the prediagnostic setting [68]. Continued research will determine whether integrating ctHPVDNA into the diagnosis, treatment, and surveillance strategies for HPV-positive OPSCC will lead to tangible improvements in patients’ treatment results.

Oncogenic HPV DNA identified in oral rinses has also been linked to treatment response and post-treatment recurrence, although with reduced sensitivity relative to ctHPVDNA and greater susceptibility to variability [69]. Bystander infections or a patient’s failure to clear viral cells post-infection are potential confounding factors, rendering this method less specific than measuring circulating tumor HPV DNA [70].

### 4.2. Droplet Digital PCR (ddPCR)

Liquid biopsies are quicker, less intrusive, and safer to obtain than tissue biopsies. The most common source used for liquid biopsies is blood [71]. Circulating tumor cells (CTCs) are released into the bloodstream by tumors, where they can be found either alone or in groups [72]. A small percentage of CTCs can infect far-off locations, migrate, and persist in plasma, potentially significantly impacting the development and spread of disease [72,73]. Blood’s extracellular vesicles (EVs) transport a variety of substances from one cell to another, including lipids, proteins, microRNAs, mRNAs, and long non-coding RNA [74]. Studies show that cancer patients’ serum contains more EVs than the serum of healthy people does. EVs are released by tumor cells into the surrounding environment, especially in hypoxic environments. These particles carry pro-migratory and inflammatory signals that stimulate the growth, invasion, and metastasis of tumors. In part, these signals are mediated by immune cells such as CD8+ T cells [71]. Mandel and Métais discovered cell-free DNA in the blood of healthy people for the first time in 1948, but today this is also known as short fragments of nucleic acid in body circulation [75]. Compared to healthy individuals, the cell-free DNA (cfDNA) of cancer patients is typically more fragmented, ranging from 160 to 180 base pairs. Since cfDNA in plasma has a half-life of around 114 min, frequent samples can be used to track the course of the disease over time [71]. cfDNA has various advantages over other liquid biopsy components, such as being simple to isolate and store and having a high sensitivity of detection [76]. However, cfDNA is unstable in whole blood, necessitating its isolation within two hours of blood collection. The National Cancer Institute (NCI) published Biospecimen Evidence-Based Practices (BEBP) in March 2020 in an effort to standardize cfDNA collection and processing across numerous organizations and institutions [77].

Quantitative PCR (qPCR), droplet digital PCR, and next-generation sequencing are the three primary methods for detecting cfDNA. Because of its picomolar sensitivity, qPCR may not be sensitive enough for low cfDNA concentrations. A housekeeping gene control is not necessary for direct and independent quantification of DNA, including cfDNA, thanks to a more recent technique—ddPCR. With an absolute quantification of cfDNA in a sample and a detection threshold as low as one copy per milliliter, it is more sensitive and repeatable than qPCR. Because a sample is divided into thousands of DNA-containing droplets, each of which is amplified separately to provide excellent detection sensitivity and specificity, ddPCR is highly effective. The third and most expensive technique, targeted gene capture (NGS), is employed to sequence cfDNA completely or partially. Despite requiring more time and resources than ddPCR, which can provide results that can be used right away, the production of NGS data can take weeks [71].

Clinical management of squamous cell carcinoma of the oral cavity (SCCOP) may benefit from the inclusion of HPV cfDNA sampling, which is currently being researched. In a study, three months after therapy, 28% of SCCOP patients had differences between HPV cfDNA detection and conventional imaging. At 12 weeks, the presence of HPV cfDNA predicted a 100% NPV and an 83% PPV of residual illness at 9 months, while imaging showed a 100% NPV and a PPV of 17.4% [78]. Based on multiple investigations, the PPV ranges from 8 to 90%, suggesting that HPV cfDNA detection is at least as effective as conventional imaging [79,80]. A different study that used two consecutive positive HPV cfDNA samples to reduce false positives found a PPV of 94% and an NPV of 100% [81].

Additionally, disease progression was predicted by HPV cfDNA detection over 0.37–12.9 months, with a median of 3.9, prior to biopsy confirmation. NGS has been used to measure the amounts of HPV cfDNA after treatment. In advanced SCCOP patients receiving chemoradiation therapy, Lee et al. discovered complete agreement between HPV E7 cfDNA detection utilizing NGS and qPCR approaches. Eighty-seven percent of 37 HPV+ patients’ responses as determined by 18F-FDG PET-CT and HPV cfDNA at 12 weeks after treatment agreed. Thirty patients had low HPV cfDNA levels and a full radiological response; six patients had increased 18F-FDG uptake but no HPV cfDNA, and their biopsies revealed no evidence of ongoing illness [82]. Using both techniques, a single patient with HPV+ liver metastases was identified. According to this study, HPV cfDNA outperforms conventional imaging in terms of positive predictive value for treatment response and disease progression. qPCR proved to be just as successful in this investigation as NGS, despite the latter’s high sensitivity. This suggests that PCR-based techniques, especially ddPCR, may be more feasible for HPV cfDNA detection in later stages because of their reduced resource requirements. It is necessary to conduct more comparative research on early-stage diseases [71].

## 5. Role and Prognostic Value of PD-L1 and Emerging Immune Checkpoint Markers in HPV-Positive Head and Neck Cancers

Immune-based therapies, particularly immune checkpoint inhibitors, have transformed the treatment landscape for HPV+OPSCC. Identifying predictive immune signatures using NGS and other high-throughput technologies has become pivotal in personalizing treatment and improving patient outcomes. These immune signatures provide insights into the tumor immune microenvironment (TIME) and help predict responses to immunotherapy. Programmed death-ligand 1 (PD-L1) serves as a critical regulator of immune checkpoints and is expressed across various immune cell types [83]. In the realm of HNC, the interaction between PD-L1 and its receptor, programmed cell death protein 1 (PD-1), instigates immune response suppression, notably impeding T cell activation [84]. It was observed that elevated expression of the immune checkpoint protein PD-L1 is more frequently found in HPV+ OPSCCs compared to HPV– OPSCCs. In some cases, this upregulation appears to result from integration of the HPV genome near the gene encoding PD-L1 (CD274) [85,86]. This inhibitory interaction compromises the innate immune system’s ability to identify and eradicate cancer cells effectively. The heightened expression of PD-L1 within the tumor microenvironment establishes an immunosuppressive barrier, facilitating cancer cells’ evasion from immune surveillance and subsequent proliferation, thereby driving tumor advancement [84]. Much effort has been spent in therapeutic settings trying to upset the PD-1/PD-L1 axis in order to renew strong antitumor immune responses. Antibodies that specifically target PD-1, like pembrolizumab and nivolumab, work by preventing the binding of PD-L1 and PD-1, which in turn enables the immune system to launch a powerful attack against cancer cells. Improved survival results in HNC patients have been demonstrated by this focused immunotherapeutic approach, signaling a paradigm shift in treatment approaches [87,88].

However, PD-L1 is more than only a therapeutic target in the management of HNC. Research has examined the predictive value of PD-L1, finding it to be a separate risk factor in oral squamous cell carcinoma. Increased expression of this gene is associated with a lower overall survival rate and distant metastases [89]. There have been contradictory reports, nevertheless, indicating that in high-risk cases of head and neck squamous cell carcinoma, increased PD-L1 expression might be associated with a longer disease-free survival [90]. PD-L1 use is hindered by its unclear role in head and neck squamous cell carcinoma subtypes, other than cancer of the oral cavity, and by the lack of information on the PD-L1 ligand in head and neck squamous cell carcinoma [91].

The increasing utilization of immune checkpoint inhibitors (ICIs) targeting PD-1 or PD-L1 in HNSCC patients will provide further insights into the reliance of HPV+ tumors on this mechanism of immune suppression. Another immune checkpoint protein, natural killer group 2 member A (NKG2A), exhibits higher expression levels in HPV+ OPSCCs with detectable HPV-specific immune responses. NKG2A is found on tissue-resident (CD103+) CD8+ T cells, which have been associated with a favorable prognosis not only in HPV+ OPSCC but also in other cancer types. While therapeutic anti-NKG2A antibodies are in the early stages of clinical development compared to those targeting PD-1 or PD-L1, they have shown promising preliminary results [9,92].

## 6. Epigenetic Markers

HPV infection, alcoholism, and tobacco use are among the factors that increase the risk of OPSCC via altering DNA methylation and other epigenetic modifications [93,94]. DNA methylation is the process of adding a methyl group to a cytosine carbon atom to create 5-methylcytosine [93]. This change in DNA structure causes changes in transcription factor activity as well as the mobility of many proteins. In head and neck cancer, hypomethylation has been connected to alcohol and tobacco use, while hypermethylation has been linked to HPV-positive tumors [95]. Furthermore, different levels of methylation have been linked to different tumor locations; particular methylation sites are associated with more rapid disease progression [93,96]. Given the increasing discovery of novel biomarkers and the necessity of applying this knowledge in clinical settings, more studies in this area are necessary.

## 7. Future Directions and Emerging Technologies

### 7.1. Development of Novel Biosensors and Biomarkers

Multiple biomarkers play a crucial role in the progression and survival of HNSCC. While the connection between p16 and HPV status with survival has been well-documented for years, it has only recently been included in staging guidelines for predicting outcomes [97]. The need for tumor biomarkers in HNSCC is becoming more urgent, both as indicators of prognosis and potential treatment targets. Just like EGFR mutations in specific lung cancers, or BRAF mutations in melanoma and certain thyroid cancers, several novel prognostic biomarkers for HNSCC have been identified through recent advancements [98]. NGS has revealed mutations such as NOTCH1, CDKN2A, and TP53, which are correlated with unfavorable outcomes [99]. In an ongoing trial, high-risk TP53 mutations (common in HPV-negative HNSCC) are being used to determine the extent of adjuvant therapy, comparing radiation alone to cisplatin with radiation [100]. The future holds promise for clinical trials that adjust treatment intensity based on additional genetic markers or signatures, leading to precision medicine strategies for HNSCC.

Markers such as tumor-infiltrating lymphocytes (TILs) and other indicators of the immune microenvironment are increasingly acknowledged as significant biomarkers in HNSCC. A study involving 464 untreated HNSCC patients found that increased levels of tumor-infiltrating lymphocytes (TILs) were linked to enhanced overall survival (OS) and disease-specific survival, even after considering various clinicopathologic factors [101]. In a group of 76 patients with advanced laryngeal SCC, CD8+ TIL counts were notably linked to the extent of clinical response to chemotherapy and predicted disease-specific survival. This suggests that TIL assessment could guide personalized treatment approaches [102]. A systematic review and meta-analysis of immune markers in HNSCC confirmed these findings, highlighting that CD163+ and M2 macrophages, along with CD57+ NK cells, are the most influential prognosticators of survival in patients with oral cavity SCC [103]. Larger studies are needed to further explore the prognostic significance of immune microenvironment signatures, which are noteworthy for both prognosis and response to immunotherapy. Nevertheless, these discoveries indicate that TIL grading could be used to tailor immunotherapy regimens, potentially enhancing the effectiveness of treatment for HNSCC patients. Future research using NGS technologies will be crucial in identifying molecular targets for HNSCC and improving prognostication.

### 7.2. Graphitic Nano-Onion/Molybdenum Disulfide Nanosheet

Molybdenum disulfide (MoS_2_) has become a hot topic in research because of its remarkable mechanical, electrical, thermal, and optical properties [104]. It is known for forming super thin nanosheets that are similar to graphene. These nanosheets are made up of layers of sulfur and molybdenum that stick together through Van der Waals interactions [105]. MoS_2_ has gained a lot of attention because of its potential applications in various fields. It can be used for chemical detection, sensing biomolecules, optoelectronics, supercapacitors, and batteries [106,107]. In the context of head and neck carcinoma, especially oropharyngeal carcinoma, MoS_2_ nanosheets have shown promise in the development of advanced biosensors. These biosensors take advantage of MoS_2_’s unique properties to detect and diagnose the disease early, which are crucial for better patient outcomes.

MoS_2_ nanosheets have been used in optochemical biosensors that can detect specific biomolecules like proteins or DNA by using their ability to quench fluorescence. For example, by combining MoS_2_ with a fluorescently labeled single-strand DNA, a highly sensitive detection platform can be created. Scientists have also combined MoS_2_ with other materials like metals, gold nanoparticles, polyaniline, polypyrrole, PEDOT, and different carbon materials [108,109,110,111]. These combinations improve electrical conductivity and sensitivity, offering cost-effective solutions for biosensors that do not require DNA labeling. Carbon nanostructures such as nanotubes, graphene oxide, and nanodiamonds have also been used to enhance the performance of biosensors for head and neck carcinoma by immobilizing DNA [112,113].

To further improve the conductivity of these carbon materials, researchers have introduced lattice defects, increased porosity, and added doping impurities [112,114,115]. Hybrid surfaces made of MoS_2_ and graphene-like structures have been developed to take advantage of these improvements, offering different functionalities based on fluorescence quenching, electrochemistry, enzyme protein-mediated electrochemistry, and calorimetry [116,117,118]. However, these approaches still face challenges, such as the need for DNA labeling and low sensitivity.

A new hybrid of nano-onions and MoS_2_ nanosheets has been developed to address the low conductivity issue of MoS_2_ in biosensors. In this study, researchers synthesized nano-onion/MoS_2_ composites by chemically combining nano-onions with MoS_2_ nanosheets. The MoS_2_ surface was modified with amines to enable the chemical’s combination with nano-onions and the adsorption of DNA [105].

This biosensor platform was designed to detect DNA from HPV-16 and HPV-18, which are important markers for early cervical cancer diagnosis and also have significance for head and neck cancers. The nano-onion/MoS_2_ biosensor, with unlabeled single-strand DNA immobilized on its surface, can detect target DNA from HPV-16 and HPV-18. By using methylene blue as a redox electronic indicator, it can detect hybridized double-stranded DNA. This upgraded platform has shown high sensitivity, as demonstrated by differential pulse voltammetry with a low detection limit [119].

The novel approach using MoS_2_ and nano-onion hybrids indeed represents a significant advancement in the development of sensitive, cost-effective, and efficient biosensors, potentially transforming the early detection and treatment of head and neck cancers. An electrochemical DNA biosensor developed using a graphitic nano-onion/MoS_2_ nanosheet composite has shown high sensitivity and specificity in detecting HPV-16 and HPV-18, enabling the early diagnosis of cervical cancer. The conductivity of MoS_2_ was improved by complexation with nano-onions, providing a suitable platform for developing effective and efficient electrochemical biosensors for the early diagnosis of various ailments, including head and neck cancers [105].

### 7.3. Potential of CRISPR-Based Diagnostics

The convergence of CRISPR (clustered regularly interspaced short palindromic repeats) technology and the field of oncology represents a groundbreaking shift in the approach to diagnosing and understanding cancers linked to HPV, particularly in head and neck cancers. With CRISPR’s unparalleled precision in gene editing, the method offers a promising avenue for early and accurate HPV diagnosis, heralding a new era in personalized medicine. This significant leap forward is not only a testament to the rapid advancements in genetic engineering but also underscores the pivotal role of CRISPR in addressing some of the most challenging aspects of cancer diagnosis. As this innovative technology continues to evolve, its application in the medical field, including the detection and study of HPV-positive head and neck cancer, represents a potential for transformative changes in patient care and treatment strategies. Additionally, the potential of CRISPR-based diagnostics is revolutionizing the diagnostic processes for HPV-related cancers, offering insights into future prospects and implications for both patients and healthcare systems [120,121,122]. The precision of this technology is pivotal in diagnostic applications, as it ensures high specificity in identifying genetic markers of diseases like HPV in head and neck cancer [123,124].

CRISPR technology offers several advantages over traditional diagnostic methods. Firstly, its high precision reduces the risk of off-target effects, enhancing the accuracy of diagnostics. Secondly, CRISPR’s efficiency in editing and detecting specific DNA sequences allows for faster diagnosis compared to conventional methods that often require more time and resources. Lastly, the adaptability of CRISPR to various genetic contexts enables its application across different types of cancers and diseases, making it a versatile tool in medical diagnostics [125,126].

CRISPR-based diagnostics are revolutionizing the field of molecular diagnostics through their simplicity and cost-effectiveness, which could democratize access to disease diagnostics. Notably, CRISPR systems, particularly the Cas12 and Cas13 enzymes, are utilized due to their nonspecific trans cleavage activity that, upon activation by the correct target, releases a fluorescent signal indicating the presence of disease markers such as HPV. This method’s adaptability and rapid response time significantly enhance the diagnostic processes, potentially improving early detection rates of HPV in head and neck cancer [124].

Despite promising advancements, CRISPR-based diagnostics face challenges, particularly in achieving high specificity without off-target effects. Continuous improvements in bioinformatics and experimental techniques are therefore essential to mitigate these risks and enhance the reliability of CRISPR-based diagnostics [124].

CRISPR technology is advancing towards clinical applications, particularly in cancer therapy, where it offers potential for precise genetic detection and treatment of HPV-driven cancers [120]. Clinical trials are already underway, evaluating the safety and efficacy of CRISPR-based therapies in humans, marking a significant step from preclinical studies to potential clinical use [127]. Ongoing research continues to explore the versatility of CRISPR technology in oncology. Studies are focusing on its application in gene editing for oncogene knockdown and engineered T cell immunotherapy, aiming to enhance the efficacy of cancer treatments [128]. The field is also witnessing an increase in clinical trials, such as those targeting PD-1 and CTLA-4 genes to improve immunotherapy outcomes [129]. These developments underscore the critical role of CRISPR in shaping future cancer treatment paradigms [127].

### 7.4. Single Gene Markers and Emerging Biomarkers

The potential of a number of other recently discovered biomarkers as prognostic indicators for HNC has been investigated in recent years. The T cell regulator SSP1, the proteasomal degradation protein PSMD14, beta tubulin isotypes, and the matrix metalloproteinase (MMP) family of enzymes that break down the extracellular matrix are a few of them [51,130]. Promising findings have been shown when beta tubulin II and III are used to predict the prognosis of taxane and cisplatin-based treatment in HNC patients [131,132]. Further research is necessary as its utility in a therapeutic environment has not yet been established. Another protein that has been linked to slow tumor progression and an advanced stage is PSMD14 [130,133]. Studies have been carried out on this protein’s possible use as a therapeutic target in the Akt pathway [133]. MMP14, MMP16, and MMP19, members of the MMP family of enzymes, have been demonstrated to have a role in immune cell infiltration in HNC [134]. However, further research be conducted to confirm the expression levels of these biomarkers, standardize their testing, and further establish their clinical value [10].

## 8. Sensitivity and Specificity of HPV Detection Techniques

HPV detection is a critical component in diagnosing head and neck cancers, particularly oropharyngeal cancers. Several methods are used to detect HPV in different samples, each with varying sensitivity and specificity, as summarized in Table 1.

Polymerase chain reaction (PCR) is one of the most widely used techniques for detecting HPV DNA. PCR’s high sensitivity (90–95%) allows it to identify very low levels of viral DNA, making it a powerful tool for initial screening. However, its specificity ranges from 80 to 90%, as it can sometimes detect clinically irrelevant HPV infections which do not cause cancer [135]. In situ hybridization (ISH) is a method for visualizing the virus within the tissue context. ISH has a sensitivity of 85–90% and a high specificity of 90–95%. This technique is advantageous since it helps confirm the presence of HPV in the cancer cells rather than in the surrounding tissues, improving diagnostic accuracy [136]. p16 immunohistochemistry (IHC) is used as a surrogate marker for HPV infection. Overexpression of p16 protein is strongly associated with HPV-positive cancers. The sensitivity and specificity of p16 IHC are both around 85–90%. While it is a useful marker, its specificity is challenging because p16 overexpression can occur in other contexts as well [137]. Serological methods for detecting antibodies against HPV proteins are less commonly used for direct diagnosis but can provide useful epidemiological data. The sensitivity of serological tests ranges from 70 to 80%, while their specificity is higher, around 85–95%. These methods are more indicative of past or present exposure to HPV rather than a direct link to cancerous cells [138]. RNA-based methods, such as RT-PCR, detect HPV RNA, indicating active viral infection. These methods have a very high sensitivity (95–100%) and specificity (90–95%), as they target viral gene expression, which correlates more directly with active oncogenic infection [139]. Next-generation sequencing (NGS) provides detailed genomic information. NGS shows the highest sensitivity and specificity, both ranging from 95 to 100%. Therefore, it is a robust but expensive and complex diagnostic tool [140,141].

## 9. Conclusions

The choice of HPV detection technique depends on the clinical context and sensitivity and specificity of each method, with PCR and ISH being common for initial diagnosis, p16 IHC serving as a useful surrogate marker, and advanced methods like RNA-based techniques and NGS providing high precision for confirming active infections and guiding treatment decisions. Each method’s sensitivity and specificity vary, influencing their utility in different diagnostic settings. Based on the sensitivity and specificity of each technique, conclusions that are more objective could be established.

Advanced technology and individualized medicine will certainly improve the future of diagnosing and treating HPV-positive HNSCC. The early diagnosis of HPV-positive HNSCC will be transformed by NGS, which detects many actionable genetic mutations concurrently, complemented by CRISPR’s unmatched gene editing specificity. These approaches will facilitate personalized therapies tailored to individuals’ tumors depending on their genetic makeup, so that treatment effectiveness is maximized while any side effects are minimized. The emergence of liquid biopsy techniques like ddPCR and circulating tumor DNA ctDNA analysis give insights into the genetic composition of the tumor without being invasive. They aid in early detection, monitoring response to therapy as well as recurrence with high precision levels. Ultimately, they may form part of routine clinical practice as they mature into advanced technologies within related medical fields, enabling disease progression and treatment effectiveness to be monitored dynamically and continuously. PD-L1 is an example of immune-related biomarkers that are increasingly gaining attention as predictors of prognosis or drug targets. The success of immune checkpoint inhibitors, such as pembrolizumab and nivolumab, at revitalizing the immune system to fight cancer underlines the potential for immunotherapy in HPV-positive HNSCC. Future studies may investigate how these treatments can be optimized and new targets within the tumor microenvironment identified to improve patients’ survival and quality of life.

Emerging markers for risk stratification include epigenetic modifications and microRNA profiles. Knowledge on specific epigenetic landscapes between HPV-positive tumors and their negative counterparts will guide future interventions that exploit these differences. Furthermore, microRNAs have been implicated in gene regulation, indicating a new direction for creating directed therapies which boost prognostic accuracy. The use of novel biosensors and composite materials like graphitic nano-onion/molybdenum disulfide nanosheets will usher in an era of early screening techniques. These platforms offer high-level sensitivity and specificity that can enable the detection of oncogenic HPV strains and related cancers in their early stages. Furthermore, the discovery of new biomarkers like SSP1, PSMD14, and various metalloproteinases would help to elucidate our knowledge of tumor biology, as well as provide possible new targets for therapy.

Future treatment for HPV-positive HNSCC depends on how fast these improvements can be successfully translated into clinical settings. There are ongoing trials and ones that will take place in the future, which could lead to confirmation regarding efficiency levels in terms of diagnostics and personalized drug delivery methods for immunotherapies. This demands a multidisciplinary approach whereby researchers, clinical workers, and technology developers come together to create platforms aimed at driving progress forward with a view to making them beneficial to patients globally. Early recognition, improved treatment, and more accurate follow-up are expected in times ahead when novel biomarkers and therapy targets will be pursued. This way, final patient results would be improved since it they enable earlier detection of HPV-related cancers, enhanced treatment, and more precise management of these malignant conditions.

## Figures and Tables

**Table 1:** Sensitivity and specificity of most common HPV detection techniques [135,136,137,138,139,140].

HPV Detection Technique	Sensitivity (%)	Specificity (%)
Serology (antibody detection)	70–80	85–95
p16 immunohistochemistry (IHC)	85–90	85–90
In situ hybridization (ISH)	85–90	90–95
Polymerase chain reaction (PCR)	90–95	80–90
RNA-based methods	95–100	90–95
Next-generation sequencing (NGS)	95–100	95–100
